# Natural Infection of the Ground Squirrel (*Spermophilus* spp.) with *Echinococcus granulosus* in China

**DOI:** 10.1371/journal.pntd.0000518

**Published:** 2009-09-22

**Authors:** Yu Rong Yang, Tianxi Liu, Xueli Bai, Belgees Boufana, Philip S. Craig, Minoru Nakao, Akira Ito, Jan Zhong Zhang, Patrick Giraudoux, Donald P. McManus

**Affiliations:** 1 Ningxia Medical University, Yinchuan, Ningxia Hui Autonomous Region, China; 2 Molecular Parasitology Laboratory, Queensland Institute of Medical Research, Brisbane, Australia; 3 School of Population Health, University of Queensland, Brisbane, Australia; 4 The Health Department of Ningxia Hui Autonomous Region, Yinchuan, Ningxia Hui Autonomous Region, China; 5 Ningxia Centre for Disease Control, Yinchuan, Ningxia Hui Autonomous Region, China; 6 Cestode Zoonoses Research Group, Biomedical Sciences Research Institute and School of Environment and Life Sciences, University of Salford, Salford, Greater Manchester, UK; 7 Asahikawa Medical College, Asahikawa, Japan; 8 Chrono-environment UMR UFC/CNRS 6249 USC INRA WHO Collaborating Centre for Prevention and Treatment of Human Echinococcosis, University of Franche-Comte, Besançon, France; Universidad Peruana Cayetano Heredia, Peru

## Abstract

**Background:**

*Echinococcus granulosus* is usually transmitted between canid definitive hosts and ungulate intermediate hosts.

**Methodology/Principal Findings:**

Lesions found in the livers of ground squirrels, *Spermophilus dauricus/alashanicus*, trapped in Ningxia Hui Autonomous Region, an area in China co-endemic for both *E. granulosus* and *E. multilocularis*, were subjected to molecular genotyping for *Echinococcus* spp. DNA. One of the lesions was shown to be caused by *E. granulosus* and subsequently by histology to contain viable protoscoleces.

**Conclusions/Significance:**

This is the first report of a natural infection of the ground squirrel with *E. granulosus*. This does not provide definitive proof of a cycle involving ground squirrels and dogs or foxes, but it is clear that there is active *E. granulosus* transmission occurring in this area, despite a recent past decline in the dog population in southern Ningxia.

## Author Summary

*Echinococcus granulosus* and *E. multilocularis* are important zoonotic pathogens that cause serious disease in humans. *E. granulosus* can be transmitted through sylvatic cycles, involving wild carnivores and ungulates; or via domestic cycles, usually involving dogs and farm livestock. *E. multilocularis* is primarily maintained in a sylvatic life-cycle between foxes and rodents. As part of extensive investigations that we undertook to update available epidemiological data and to monitor the transmission patterns of both *E. granulosus* and *E. mulilocularis* in Ningxia Hui Autonomous Region (NHAR) in northwest China, we captured small mammals on the southern slopes of Yueliang Mountain, Xiji, an area co-endemic for human alveolar echinococcosis and cystic echinococcosis. Of 500 trapped small mammals (mainly ground squirrels; *Spermophilus dauricus/alashanicus*), macroscopic cyst-like lesions (size range 1–10 mm) were found on the liver surface of approximately 10% animals. One of the lesions was shown by DNA analysis to be caused by *E. granulosus* and by histology to contain viable protoscoleces. This is the first report of a natural infection of the ground squirrel with *E. granulosus*. We have no definitive proof of a cycle involving ground squirrels and dogs/foxes but it is evident that there is active *E. granulosus* transmission occurring in this area.

## Introduction

The canid adapted intestinal tapeworms, *Echinococcus granulosus* and *E. multilocularis* are important zoonotic pathogens that cause serious disease in humans [1]; both are endemic to Ningxia Hui Autonomous Region (NHAR) in northwest China [2],[3]. *E. granulosus* can be transmitted through either sylvatic cycles, involving wild carnivores and ungulates; or via domestic cycles, usually involving dogs and farm livestock. A common source of infection for dogs is hydatid infected offal from sheep, which often harbour the common G1 genotype (sheep-dog strain) responsible globally for most cases of human cystic echinococcosis (CE) [1],[4]. *E. multilocularis* is primarily maintained in a sylvatic life-cycle between foxes and rodents, with human infections considered as a relatively rare accidental event caused by spill-over from the wildlife cycle in European countries [5]. Synanthropic transmission cycles are believed to be responsible for the high prevalence of human alveolar echinococcosis (AE) in Alaska and on the eastern Tibetan Plateau, whereby domestic dogs predating on rodents in and around villages are considered to be the primary source of infection causing human AE [6],[7].

A report of *E. granulosus* in plateau pika (*Ochotona curzoniae*) in Qinghai Province [8] appears retrospectively almost certainly to be due to *E. shiquicus*, a new *Echinococcus* species described in 2005 that infects Tibetan foxes (*Vulpes ferrilata*) on the Tibetan Plateau [9]. Work in the 1980s in NHAR indicated that the transmission modes for co-hyperendemic AE and CE involved domestic dogs/livestock (mainly sheep) for CE and foxes/rodents for AE [10].

**Figure 1:**
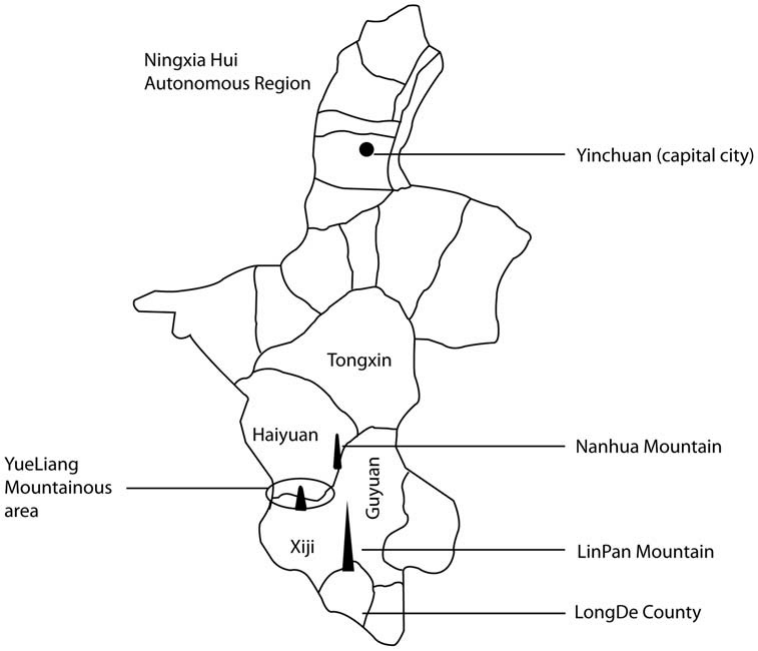
Map of the Yueliang Mountain area in Southern NHAR, China.

Extensive investigations that we undertook in 2001–2007 to update available epidemiological data and to monitor the transmission patterns of both *E. granulosus* and *E. mulilocularis* in NHAR, indicated that owned dogs were a risk factor for human AE (involving a dog/rodent cycle) as well as CE (involving a dog/domestic livestock cycle) [3],[11]. An increase in susceptible rodent populations due to deforestation and over use of farmland for agriculture have been emphasised as important zoonotic risk factors for human AE in NHAR and in other Chinese settings [11],[12]. As part of these ongoing studies, we captured small mammals on the southern slopes of Yueliang Mountain, Xiji County (Figure 1) (E, 105°64′–105°89′; N, 36°03′–36°18; altitude ranging from 2000–2200 m) in July, 2007. This is an area known to be co-endemic for both human AE and CE [3], and where high seroprevalence for echinococcosis among village-children has been recorded [13]. Of 500 trapped small mammals (mainly ground squirrels; *Spermophilus dauricus/alashanicus* referred to also as *S. dauricus*, *Myospalax fontanieri* and *Mus musculus*), macroscopic cyst-like lesions (size range 1–10 mm) were found on the liver surface of approximately 10% animals. Lesions were subjected to molecular genotyping and histopathological examination. None were attributable to *E. multilocularis* but one lesion was identified unambiguously as *E. granulosus*, subsequently shown by histology to contain viable protoscoleces. This is the first report of a natural infection of the ground squirrel with *E. granulosus*.

## Materials and Methods

This study was reviewed and approved by the Ethics Committee of Ningxia Medical University. All small mammals were humanely euthanized soon after being trapped. Animals were identified, dissected and the obtained livers fixed in absolute ethanol for DNA analysis and histology.

Prior to histopathology, involving sectioning, haematoxylin/eosin staining and microscopic examination by standard procedures, liver lesions were transported to the Cestode Zoonoses Laboratory (University of Salford, U.K.) for molecular genotyping. Genomic DNA was extracted from these lesions using the DNeasy tissue kit (Qiagen, Hilden, Germany) according to the manufacturer's instructions and used as a template for the amplification of a fragment within the mitochondrial 12S rRNA gene [14],[15]. Amplified cestode-specific DNA was gel purified using the PureLink™ quick gel extraction kit (Invitrogen, Paisely, U.K.) and commercially sequenced (Cogenics, Takeley, U.K.). The identity of one of these samples was confirmed in another laboratory (Department of Parasitology, Asahikawa Medical College, Asahikawa) by partial sequencing of the mitochondrial *cox1* gene as described [16].

## Results

Comparison of the generated sequence data with those held on the NCBI database (www.ncbi.nlm.nih.gov) through the use of BLAST program revealed one sample had 100% homology (254 bp) with the mitochondrial 12S rRNA gene of *E. granulosus* genotype G1 (common sheep-dog strain) (accession nos. DQ408422, AF297617, AB031350, AB024515). Compared with previously published gene sequences (AF297617, *E. granulosus* G1 (common sheep-dog strain); AB 018440, *E. multilocularis*), *cox1* sequence (789 bp) for the sample was nearly identical to that of the published *E. granulosus* G1 sequence with the exception that three transitional changes were present at positions 243 (G/A), 530 (C/T) and 594 (T/C) for the isolate. The sequence shared only 80% identity with the published *E. multilocularis cox1* gene sequence.

**Figure 2:**
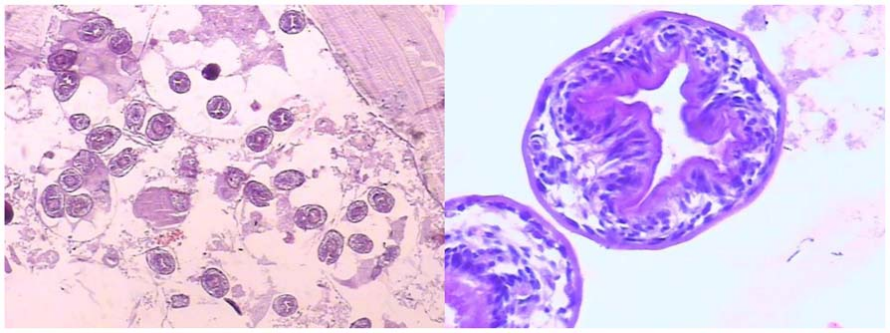
Histopathologic section (haematoxylin and eosin stain) of the liver lesion from a ground squirrel (*Spermophilus dauricus/alashanicus*). Left panel shows the typical appearance of a fertile *Echinococcus granulosus* cyst with laminated and germinal layers, brood capsules and numerous viable protoscoleces; right panel is an enlarged part of the section showing two viable protoscoleces (×1000).

Subsequent histological examination of this ground squirrel liver lesion revealed the presence of a thick laminated layer, thin germinal layer and presence of brood capsules containing viable *E. granulosus* protoscoleces (Figure 2).

## Discussion

There are numerous previous reports of small mammal species infected with *E. multilocularis* in China and Europe [12],[17]. It is well accepted that microtine rodent species are the main reservoir hosts of *E. multilocularis*, though other rodent groups and even lagomorphs (hares and pikas) may also be naturally infected [6],[18]. However as far as we know, apart from experimental infection of rodents using either protoscolex or oncosphere injection [19],[20], or oral administration of viable eggs [21], this is the first report of a rodent species naturally infected with the metacestode stage of *E. granulosus*. Other non rodent small mammals harbouring lesions of *E. granulosus* (identified morphologically) have been described in hares in Argentina [22] and rabbits in Australia [23],[24]. The current observation has shown, for the first time, the rodent, *Spermophilus dauricus*, is susceptible and can be infected naturally with *E. granulosus* that can become viable, producing fertile cysts.

Land cover in the southern mountainous areas of NHAR has undergone important changes since the second half of the 20th century. The area was largely deforested in the 1970s–80s, and in the rolling hills around the southern Liupan Shan, total tree clearance was completed in the mid 90s. Now, the landscape consists entirely of fields for production of wheat, potatoes, beans, alfalfa, etc. During the late 1970s valleys and lower slopes were generally used for agricultural crop production while the upper slopes and hill tops were reserved for grazing. At the time there were no livestock restrictions and grazing pressure was intense. In the late 90s, massive reforestation campaigns carried out to prevent soil erosion led to extensive re-planting of trees and restrictions in sheep numbers allowable per family. The landscape changes had a subsequent major effect on small mammal communities [25] and may have played a role modifying cestode transmission patterns involving small mammals. For instance the opening of the landscape during the deforestation process may have increased the area of habitats favourable to *Spermophilus dauricus* [25]. On the other hand, the dog population has been recovering after the banning of indiscriminate rodenticide use in NHAR from 2002 [12]. The high susceptibility of various host species present together with high parasite prevalence may have increased the infection of definitive and intermediate hosts for both *E. granulosus* and *E. multilocularis*. It is possible that free roaming dogs not only could get infected with *E. granulosus* after feeding on discarded sheep offal containing larval *E. granulosus* but also perhaps through predation on *Spermophilus dauricus*. This large rodent species is one of the commonest in the area and largely occurs in fields, fallows and in the early stages of re-forestation. The red fox (*Vulpes vulpes*) which mostly feeds on small mammals is also a potential candidate for *E. granulosus* transmission in this area of NHAR since this canid species has been shown to be susceptible by experimental infection [11], although it usually harbours smaller worm burdens than dogs, and it has been found naturally infected in Australia and Europe [26]–[31].

Although we have no definitive proof of a cycle involving ground squirrels and dogs/foxes, it is clear there is active *E. granulosus* transmission occurring in this area, despite the recent past decline in the dog population in southern Ningxia [3],[12]. Possible misidentification of morphological specimens of *Echinococcus* obtained from small mammals may have occurred in the past [10]. Therefore, in further epizootiological surveillance of echinococcosis, it would be useful to apply DNA typing of metacestodes from small mammals and copro-DNA techniques [32] for unambiguous identification of fox or dog infections in order to provide accurate baseline data on transmission and to inform a model [33],[34] for future integrated control options.
